# Influence of Depression and Personality on Social Functioning in Older Adults

**DOI:** 10.1093/geroni/igab046.3120

**Published:** 2021-12-17

**Authors:** David Freedman, George Lederer, Lauren Atlas, Rachel Waldman, Jennifer Ho, Helene Geramian, Dimitry Francois, Richard Zweig

**Affiliations:** 1 Lenox Hill Hospital, Brooklyn, New York, United States; 2 Jacobi Medical Center, Scarsdale, New York, United States; 3 New York-Presbyterian/Columbia University Irving Medical Center, NEW YORK, New York, United States; 4 Weill Cornell Medicine, New York, New York, United States; 5 Yeshiva University, Bronx, New York, United States; 6 NY, White Plains, New York, United States

## Abstract

Among older adults there is significant comorbodity between depression and personality pathology and both are associated with poorer social functioning. Personality pathology is associated with greater prevalence, poorer recovery, and a higher likelihood of recurrence of depression in older adults. This study is a secondary analysis examining the relationships between personality traits associated with personality pathology (i.e. high neuroticism and low agreeableness), depression, and social functioning across older adults surveyed in primary care and psychiatric inpatient settings (N = 227). Individual variable as well as interaction models were examined. Higher neuroticism (FChange [1,217] = 40.119, p < .001), lower agreeableness (FChange [1,217] = 20.614, p < .001), and clinical status (i.e. primary care vs. psychiatric inpatient) (FChange [1,217] = 19.817, p < .001) were associated with poorer social functioning. Clinical status moderated the relationships between neuroticism and social functioning (B = -.0147, p = . 0341) and between agreeableness and social functioning (B = .0268, p = .0015). Interaction effects were not observed between neuroticism and depression or agreeableness and depression as they relate to social functioning. However, depression severity was observed to mediate the relationship between neuroticism and social functioning [Indirect effect = .0212, 95% CI = .0141, .0289]. These findings highlight the importance of accounting for depression and clinical status in the assessment and treatment of older adults with personality pathology. Findings warrant future research focused upon mechanisms through which personality pathology and depression influence functional status in older adults.

